# Notice to All Interested

**Published:** 1905-12

**Authors:** Louis Jack

**Affiliations:** President


### Editorial.

---

### NOTICE TO ALL INTERESTED.

At a meeting of the Board of Directors of the International Dental Publication Company, held October 20th, it was resolved to discontinue the publication of the International Dental Journal with the December number.

Louis Jack,
*President.*

The foregoing important notice from the President of the International Dental Publication Company is self-explanatory and requires no further comment. This journal closes its eighteen years of independent life with this number.

